# Development of an Optimal Model of Combined Radiation and Biological Lesions

**DOI:** 10.1155/2022/9433032

**Published:** 2022-09-08

**Authors:** Timur R. Gaynutdinov, Konstantin N. Vagin, Yagafar M. Kurbangaleev, Kamil T. Ushmukhametov, Farit Kh Kalimullin, Vanentina A. Guryanova, Ilnar R. Yunusov, Aleksey V. Frolov, Fanil R. Vafin

**Affiliations:** Federal Center for Toxicological, Radiation and Biological Safety, Nauchnyi Gorodok-2, Kazan 420075, Russia

## Abstract

Since the search for the effective medication in combined lesions includes the selection of an optimal experimental model for such injuries, there is actually a study aimed at developing an optimal model of combined radiation-biology (*Pasteurella*) lesions. The pathogen *Pasteurella multocida* (as one of the most frequent pathogenic agents involved in both isolated and combined radiation-biology lesions of agricultural animals) was used as a model of a biological agent to reproduce experimental biological research. We employed the “Chinchilla” rabbits of 2.5–3.0 kg body weight as a biological model for doing combined radiation *Pasteurella* lesion. When determining the optimal model of combined radiation-biology (*Pasteurella*) lesion, we consider that in the joint action of various pathological agents on the organism, there is a synergistic effect of explosion agents, previously specifying minimal doses of external *γ*-radiation and pasteurellosis pathogen that in the joint action of nonfatal doses would be lethal. The first stage of the experiments determined the minimal doses of gamma rays and pasteurellosis pathogen that in joint action causes combined radiation-biology pathology. We examined 66 rabbits divided into 11 groups of 6 animals each to determine minimal doses of infectious agent-pasteurellosis pathogen. The animals of the first 9 groups were given subcutaneously *Pasteurella* species at doses 1·10^9^, 1·10^8^, 1·10^7^, 1·10^6^, 1·10^5^, 1·10^4^, 1·10^3^, 1·10^2^, and 1·10^1^ of microbial cells per animal of 0.3 ml suspension in volume; the 10th group of animals were given saline solution; the 11th served as a biological control group. In determining the minimal doses of gamma rays, we conducted experimental tests on 36 rabbits, which have been exposed to external *γ*-radiation in the “PUMA” system with a ^137^Cs radiation source of the exposure dose of 5.38 R/min at doses 2.0, 4.0, 6.0, 8.0, 10, and 12 Gy. To specify the optimal model of radiation-pasteurellosis lesion, we used the rabbits subjected to a combined radiation-biology effect using minimal doses of gamma rays and pasteurellosis agent, leading to a lethal effect during their complex action. The researches revealed that 50% of the death of rabbits infected with pasteurellosis occurs using *Pasteurella* at a dose of 3.7·10^4^ microbial cells per kilogram (*LD*_50_ = 3.7∙10^4^ m.c./kg), and 50% of radiation death in rabbits occurs when irradiated their gamma rays at a dose of 8.0 Gy (*LD*_50_ = 8.0 Gy). The combined effect of nonlethal doses of the studied agents in the indicated doses on rabbits led to the aggravation of the course of radiation and pasteurellosis infection, causing the death of animals from combined radiation-pasteurellosis pathology. The model combined radiation-pasteurellosis disease ran its course rapidly, and the animals died 3 to 6 days after the onset. The autopsy of the animals that died from acute radiation-pasteurellosis pathogen had found swelling of the subcutaneous tissue in the pharynx and intermaxillary space of the neck, hyperemia, lymphoid nodular hyperplasia, numerous hemorrhages on the serous and mucous membranes and in the tissues of the parenchymal organs, serous or serous-fibrinous exudate, and in the chest and abdominal regions, pulmonary edema. The research stated that gamma radiation of rabbits at a dose of 8.0 Gy conducted before exposure with *Pasteurella* at *LD*_50_ (3.7·10^4^ m.c./kg) declined the course of the pasteurellosis process, facilitated its generalization, and fastened the death of animals. Combined radiation-pasteurellosis infection ran its course rapidly, and the animals died within 3 to 6 days after the onset of the disease. The autopsy showed the pathologicoanatomic factors of the acute pasteurellosis: swelling of the subcutaneous tissue, purulent-catarrhal bronchopneumonitis, and pulmonary edema.

## 1. Introduction

Potential violation of rules leading to technological accidents and nuclear explosions come amid combined action of thermal, chemical, physical, and biological damage to human and animal organisms [1, 2].

A nuclear explosion resulted in simultaneous exposure to radiation of animals and, in a short time, within the first four days, a radiation disease, the course of which depends on the type of radiation, power, amount of the dose received, external factors, and individual characteristics of an organism [3, 4].

Radiation action on the organism inhibited bone marrow hemopoiesis and emptied central and peripheral immune organs, causing the growth of autoimmune processes. The therapy of acute radiation disease is a preparation based on chemical and biological substances [5–8].

A release of radioactive elements into the atmosphere results in their spread in large territories where agribusiness centers containing animals are situated.

Plenty of diseases of microbial and viral origin that cause colossal economic damage occur in epizootiology. The most dangerous diseases are those characterized by the carriage of virulent strains of microorganisms, one of which is pasteurellosis caused by *Pasteurella multocida*.

Pasteurellosis is an infectious disease that affects domestic and wild animals. There are reports [9, 10] of the possibility of spreading the disease in humans. The diseases are hyperacute, acute, subacute, and chronic; the disease has the symptoms of septicemia and hemorrhagic inflammation of intestinal mucosa and respiratory tract. The incubation period is from several hours to three days; the mortality rate is from 10 to 80%.

The infected, recovered, and carriers of infectious agents (“Pasteurellocarriers”), which isolate the pathogen by running from the nose, breath, saliva, and feces, determine the basic reasons for the disease emergency [11].

“Pasteurellocarriage” in the infection lasted for over a year. Many authors consider pasteurellosis related to the importation of animals that carried *Pasteurella* from contaminated households. “Pasteurellocarriers” from prosperous households cause the spontaneous emergency of pasteurellosis free of external inflammation that occurs against the impact of various adversities on the animals, one of which is radiation stress [12].

Thus, the emergencies of nuclear explosion result in the reduction of the immunity in the animals and pasteurellosis coupled with radiation disease. The course of the disease, pathogenesis, and outcome of the lesions have their own characteristics, as radiation and biological factors simultaneously affect the body. Accordingly, the purpose of the research was to develop the model of radiation-pasteurellosis lesions of the organism since the risk of both isolated and combined lesions of agricultural animals and humans with ionizing radiation and biological agents remains.

## 2. Materials and Methods

The research on the modeling of radiation-pasteurellosis infection included a number of phases. The first phase of the studies involved defining the optimal conditions for reproduction of pasteurellosis lesions of the organism in accordance with the target set. The isolate of the culture *Pasteurella multocida* extracted from the pathological material of a calf from a contaminated household was used as an agent causing infectious lesions.

We studied the morphological and cultural characteristics of the selected strain in accordance with generally accepted methods in microbiology.

In total, 66 Chinchilla rabbits of 2.5–3.0 kg were used as a biological model to reproduce the experimental pasteurellosis infection.

Before the experimental modeling of pasteurellosis, we determined the virulence of the pathogen used as a test strain for reproducing pasteurellosis infection. *Pasteurella* were cultivated in Petri dishes in meat infusion agar (MIA) with added 10% horse blood serum. The growing cultures were washed off with saline of 7.2–7.4 pH.

The smears were stained according to Gram. For this purpose, we applied a sterile drop of saline solution to glass slides contained in alcohol-ether, then placed the analyzed culture from agar in a loop, and mixed thoroughly. The smear was dried in the air, fixing over a burner flame. Gentian violet was applied to fixed smears for 2 min, then to Lugol's solution for 2 min, and ethyl alcohol from 30 sec to 1 min and washed off with distilled water. The strained smears were air-dried and immersed microscopically at 90-fold magnification.

To determine *LD*_50_ of *Pasteurella*, we divided the animals into 11 groups of 6 each. The animals of the 10th group (control) were given the saline solution; the 11th served as a biological control group. The experienced animals were given subcutaneously a relevant solution of washing away cultures of 0.3 ml/head in one piece 10^9^ and diluted in the ratios 1 : 10, 1 : 100, 1 : 1000, 1 : 10000, 1 : 100000, 1 : 1000000, 1 : 10000000, and 1 : 100000000.

The scientists observed the animals for 10 days registering the dead and survived animals. The calculation of *LD*_50_ was performed by a Pershin method using the following formula:

(1) $$\begin{array}{c} LD_{50}=\frac{\sum \left[\left(aa+bb\right)\left(m-n\right)\right]}{200}, \end{array}$$

where (*a*+*b*) is total related doses; (*m*−*n*) is the difference between mortality rate and two subsequent doses.

To determine the optimal doses of gamma rays, the scientists examined rabbits irradiated on the PUMA gamma ray system with a ^137^Cs radiation source of the exposure dose of 5.38 R/min at doses of 2.0; 4.0; 6.0; 8.0; 10.0 и 12.0 Gy. The experiments used 42 Chinchilla rabbits divided by analogy into 7 groups of 6 animals each. The animals of the 1st group were irradiated at a dose of 2.0 Gy, the 2nd, 4.0 Gy; 3rd, 6.0 Gy; 4th, 8.0 Gy; 5th, 10.0 Gy; 6th, 12.0 Gy. The animals of the 7th group were not irradiated and infected—they served as the biological control group.

The animals were daily observed, noting the state of the visible mucous membranes, coat, behavior reactions, food and water consumption, physical activity, the course and outcome of acute radiation disease, pathological changes in organs and tissues of the dead animals, and overage life.

The next stage of the research work involved the studies of the effect of gamma irradiation on the course and outcome of radiation disease on combined exposure to radiation and infectious disease.

The studies were carried out on 25 adult rabbits of Chinchilla of both sexes with a body weight of 2.5–3.0 kg divided into 4 groups by analogy of 5 animals in the 1st, 2nd, and 4th groups and 10 animals in 3d. While animals of the 1st group were exposed to the isolated effect of gamma rays at a half-lethal dose (8.0 Gy), those in the 2nd group were exposed to the isolated effect of the pasteurellosis causative agent at a half-lethal dose (3.7·10^4^ m.c./kg). Animals of the 3d group were subjected to combined radiation and biological action (CRBA); the 4th group was not irradiated and infected and served as a group of biological control.

An isolate *P. multocida* was obtained in the contaminated household with titrated virulence *LD*_50_ = 3.7∙10^4^ m.c./kg. Acute radiation disease of rabbits was caused by gamma irradiation on the PUMA gamma ray system with an exposure dose of 5.49 R/min (1.42·10^−4^ C/kg).

The CRBA was implemented by irradiation of 10 rabbits on a PUMA gamma ray system in a half-lethal dose of 8.0 Gy and the animals were immediately infected with a virulent strain of the pathogen by intravenous administration of 1.0 ml of 18–24-hour *Pasteurella* broth culture in a pretitrated dose of 3.7·10^4^ m.c./kg.

The irradiated infected with pasteurellosis causative agent and exposed to combined radiation and biological actions rabbits were observed for 30 days after isolated and combined exposure, studying the clinical manifestations of acute radiation disease, pasteurellosis, CRBA, the course and outcome of the disease, and pathological changes in the organs and tissues of the dead animals.

Acute radiation disease (ARD) and pasteurellosis were diagnosed based on the complex of clinical, pathologicoanatomic data and results of bacteriological research of pathological material with compulsory biological analyses of the isolated culture on white mice.

## 3. Results and Discussion

The results of microbiological studies showed that the growth of *Pasteurella* during the first 24–48 hours of incubation on nutrient broth was accompanied by a slight homogenous hazing with specific mucous productions in the bottom of a test tube by the 4-5th day of the research rising in the form of an unbreakable braid. On solid serum-supplemented medium, *Pasteurella* increased in the form of small transparent round colonies with straight edges; in further cultivation, the colonies acquired a gray-white color; when rising on blood agar, the colonies did not form a hemolysis zone.

In microscopy of smears, *Pasteurella* were of the form of ovoids or short rods with rounded ends and noticeable bipolarity around which a transparent capsule is visible, and when stained according to Gram, Gram-negative short ovoid rods with rounded ends.

Table 1 presents the titration results for *P. multocida* Pasteurella.

The data show that the administration of a suspension of *Pasteurella* of 10^7^–10^8^ m.c. caused the death of all rabbits, while administrating 10^4^ of suspension led to the death of 50% of animals, and when 10^1^-all animals survived. Therefore, *LD*_50_ of the *P. multocida* culture for animals is in the range of 10^4^–10^5^ m.c./kg.

The research work reports that the death of infected rabbits at a dose of 3.7·10^4^ m.c./kg occurred on the 7–10th day after the injection of the culture suspension with symptoms of the disease characteristic of pasteurellosis infection, namely, depression, refusal of feed, increased thirst, weakness, the lack of any reaction to the environment, muscle tremors, swelling of the subcutaneous tissue in the head, neck, dewlap, and scapula.

The results of the analyses of the course and outcome of the disease in rabbits irradiated with *γ*-rays ^137^Cs in the dose ranging from 2.0 to 12.0 Gy with a step of 2.0 Gy, presented in Table 2, showed the rate of manifestation of clinical and the emphasis of pathological signs depends on the dose of gamma irradiation.

The data in the table indicate that the irradiation dose of 12.0 Gy caused an extremely severe degree of ARD (*LD*_100/30_), which was characterized by excitement, increased heart rate and respiration, increased intestinal peristalsis, and body temperature to 41°С. The animals mostly lay down and did not take feed but drank water. Some animals had signs of bloody discharge from the nostrils, anemia of the conjunctiva of varying degrees, and drying crusts in the corner of the eyes. Diarrhea and small punctate hemorrhages on visible mucous membranes were found almost in all animals. We registered the death of all animals on the 5th, 7th, 9th, 11th, 12^th^, and 14^th^ days. The average life expectancy was 9.7 days.

A dose of 10.0 Gy caused in rabbits acute radiation disease (*LD*_83.3/30_) during the first day of which we noted excitation of the animals, in some rabbits–muscle tumors, then depression, and hypodynamia. The pulse and respiratory rate increased, and the body temperature rose to 40°С. The animals' health status remained depressed; they were reluctant to take feed, drank water, and had liquid feces, and some animals had short-term diarrhea. On the 4-5th days of the experiment, the general condition improved, the temperature decreased, and appetite and physical activity developed. On the 8th day, the height of radiation disease is accompanied by a rising of the body temperature to 40°-41°С, purulent discharge from the nose, liquid feces, depression lying, less feed and water intake, disheveled coat, and dark brown drying crusts in the corners of the eyes and nasal passages. The death of animals in this group was observed on the 8th, 10th, 14th, and 16th (2 heads) days. The average life of the dead animals was 12.8 days.

Pathological changes in dead animals with acute radiation disease (ARD) of an extremely severe degree were similar to those in ARD of a severe degree and developed in the form of dystrophic changes in the heart muscle, hypoplasia of hematopoietic tissue, in some cases marked, hemorrhagic diathesis, a decrease in the spleen, edema and flabbiness, and liver degeneration. Extensive hemorrhages were found in all organs and tissues of the dead animals.

Irradiation of rabbits at a dose of 6.0 and 8.0 Gy caused a moderate radiation disease; the research registered the death of 2 animals on the 24th and 26th day at a dose of 6.0 Gy and of 3 animals on the 23rd, 24th, and 25th day of the experiment at a dose of 8.0 Gy with an average life 25 and 24 days, respectively.

External irradiation at doses of 2.0 and 4.0 Gy caused mild radiation disease in rabbits, characterized by an increase in pulse and respiration in individual animals; by the 20th day, the animals weakly took feed, drank water, lay more than usual, and had short-term liquefaction of feces, with lameness noted in some animals, which gradually returned to normal. However, on the 27th day, we recorded the death of the 2nd group (irradiated at a dose of 4.0 Gy).

The studies carried out have shown that *LD*_50/30_ is a *γ*-irradiation dose of 8.0 Gy, which will be used as a model in the development of the GRBA.

The results of dynamic observations of irradiated (1st group), infected with *Pasteurella* (2nd group), and irradiated and infected with *Pasteurella* (3rd group) rabbits showed that the course of ARD, pasteurellosis, and combined radiation-pasteurellosis lesions had significant differences depending on the type of exposure of pathological agents (Table 3).

The course of ARD in rabbits irradiated in *LD*_50/30_ was of a mild form that, in the beginning, proceeded without marked clinical signs. The latent period lasted 10 days. The height of radiation disease began gradually. This period included a partial refusal of feed, hyperemia, soreness of the skin, and anemia of visible mucous membranes, sometimes followed by hemorrhages. In some animals, diarrhea was periodically noted with a slight admixture of mucus in feces. The lesions of the blood system, gastric, and respiratory tract in some animals progressed and 2 rabbits of 5 irradiated died from ARD on the 23rd and 25th day after irradiation.

The course of pasteurellosis infection in animals of the 2nd group infected with *P. multocida* at a dose of *LD*_50_ (3.7·10^4^ m.c./kg) was subacute and characterized by slower development of signs of fibrinous pneumonia. Soreness of the chest appeared on palpation; the type of breathing was abdominal. The disease was followed by severe painful cough, serous or mucous discharge from the nose, often with blood; conjunctivitis, hemorrhages on the skin, and symptoms of dysphoria (coprostasis or diarrhea) were often observed during the experiments. The body temperature rose to 41–41.5°С. With increasing signs of cardiac weakness and breathing difficulties, some animals (3 out of 5) died after 26–28 days.

Postmortem autopsy of dead animals revealed fibrinous pneumonia, hepatization, and marbling of the lung tissue, catarrhal gastroenterocolitis.

The course of combined radiation-pasteurellosis infection in rabbits had acute thoracic form. At the same time, we observed in animals depression and weakness; they lay more, rose reluctantly, and had quickened pulse and reddish dry mucous membranes. We noted refusal to feed, increased thirst, and rapid and labored breathing. The temperature increased to 41–42°С, and edema was noted in the region of the pharynx and neck, causing the development of pharyngitis. A reddish-bluish coloration of the skin of the ears appeared. Sometimes, there was a severe cough and coprostasis, often rotated to diarrhea with blood or mucus.

The combined radiation-pasteurellosis disease ran its course rapidly, and the animals died 3–6 days after the onset of the disease. Autopsy of animals that died in the acute course of radiation-pasteurellosis pathology revealed edema of the subcutaneous tissue in the pharynx and intermaxillary space of the neck, hyperemia, and enlargement of lymph nodes, numerous hemorrhages on the serous and mucous membranes and in the tissues of the parenchymal organs, serous or serofibrinous effusion in the chest and abdominal regions, and pulmonary edema.

To confirm the diagnosis of pasteurellosis with radiation biological lesions to the body, we carried out bacteriological studies in parallel to detect the pathogen (excretion of *Pasteurella*), determine virulence, and identify and differentiate the isolated pathogen.

For bacteriological analyses, we took the blood of animals, pieces of spleen, liver, kidneys, and injured parts of the lungs, lymph nodes, and tubular bone. The studies were carried out by microscopy of smears or smears-prints from the affected organs. For the isolation of *Pasteurella*, suspensions of samples from parenchymal organs and bone marrow were plated on nutrient media and contaminated the white mice.

Microscopic examination of blood smears and smears-prints from tissues of parenchymal organ, stained according to Gram, revealed short Gram-negative with rounded ends ovoid bipolar rods. The smears prepared from broth and agar cultures revealed small coccoid bacilli-bipolar typical of *Pasteurella*.

During the research work, we used the principle of consistent application of the method of scientific knowledge: from the results of the analysis of information sources to substantiate the relevance and clarification of tasks through a comparative synthesis of experimental modeling data to obtain an adequate model and the subsequent application of scientific methods (radiobiological, epizootic, clinical, morphological, and statistical) in isolated and combined radiation and biological lesion. In particular, we used laboratory animals exposed to radiation and infectious agents.

Pathogenic *Pasteurella* plays an important role in the occurrence of pasteurellosis in agricultural animals, persists a long time in the body not only of healthy animals who have been ill and in contact with them and in the body of synanthropic animals and birds, creating a kind of epizootic focus [13, 14]. In this regard, “*Pasteurella* carriage” should be considered a dangerous source of the causative agent of the disease, which, in the presence of susceptible animals, will produce an epizooty. One of the strongest stress factors is *γ*-irradiation [15, 16].

The scientists of the Kazan Scientific Research Veterinary Institute have recently done a large experimental work on radiobiology, particularly acute radiation disease and combined lesions of animals [17, 18].

The term *radiation disease* did not exist until 1945; it was common to talk about radiation reactions occurring in sick people under the influence of radiation treatment of malignant tumors when it was necessary to irradiate the body with large doses of X-rays. The tragedy that occurred because of the bombing of the Japanese cities of Hiroshima and Nagasaki on August 6 and 9, 1945, led to many patients with isolated and combined factors. In response to the study of the clinical picture and pathogenesis of the disease, acute radiation disease was isolated as a separate nosologic unit [19, 20]. However, in a natural-industry-related field, an isolated course of radiation disease is rare; mainly, combined lesions are recorded [21].

The study of the combined radiation lesions in peacetime in practical conditions is impossible due to the absence of this type of injury, so researchers need to refer to the method of modeling CRL on laboratory animals. Given that in the case of the use of nuclear weapons, up to 80–85% of those affected with CRL are expected, treating the pathology takes one of the leading places [22, 23]. In the immediate period after exposure to the damaging factors of a nuclear explosion or accident, it is precisely the timely complete medical manifestations that determine the survival prognosis [24].

Combined radiation lesions are a multicomponent pathological process progressing with simultaneous or sequential exposure of the body to ionizing radiation and damage effects of radiation-free etiology [25]. The following mechanical, thermal, chemical, and biological factors are distinguished as damaging factors of a nonradiation nature [26].

CRL has a number of features, of which the most characteristic is the presence of signs of two or more pathologies, because of which a specific clinical picture of radiation and traumatic symptoms is formed [27]. Another feature of the CRL is a predominance of the leading components in a specific period of the development. Finally, the third feature is this type of pathology develops a process, the peculiarity of which is the interrelation and interdependence of radiation and nonradiation lesions, which is not observed with isolated radiation lesions or another type of nonradiation component of CRL. The interaction of CRL components, injuries, burns, or other factors resulted in the involvement of a greater number of body systems in the pathological process and increased the severity of their dysfunctions. The complex of signs indicating a more severe course of each of the CRL components is called the mutual burden phenomenon [28]. As the lesions are summed up in a single organism, the general health state of the affected person is clinically worsening. As a consequence, there is an increase in mortality rate in CRL compared with mortality in each of its components separately.

Ionizing radiation is a specific mandatory component of CRL, which generally determines the characteristics of the course of the pathological process.

The radiation component of CRL firstly inhibits the function of hematopoiesis, causing bone marrow aplasia. The peripheral blood records the signs of pancytopenia, and it is clinically appeared by the development of endotoxicosis and infectious and hemorrhagic syndromes [29].

Developing immunodeficiency after radiation exposure (secondary immunodeficiency of pancytopenic type) leads to infectious complications. A decrease in antimicrobial resistance occurs because of the death of highly radiosensitive lymphocytes, dysfunction of macrophages, and death of leukocytes. The source of infectious complications, in this case, is its own microflora, vegetating in the lumen of the gastrointestinal tract, respiratory tract, skin, and mucous membranes [30].

When modeling radiation biological pathology, we proceeded from the fact that under the action of two pathogens, there is an aggravation and complication in the course of the combined disease.

The development of the phenomenon of mutual burdening, as a feature of the course of CRL, is shown in research works of various authors, so in the case of a combined exposure, the survival rate is halved compared to an isolated disease [31].

We conducted experimental work on rabbits to create an optimal testing model of severe combined radiation biological lesions (CRBL). Our task was to make an adequate choice of the nature of radiation exposure and biological damage. According to modern concepts, in order to develop severe CRL for the injured parts, radiation exposure is necessary, causing the development of a marrow form of ARD of moderate severity, combined with pasteurellosis infection. For these reasons, we used an indicator *LD*_50_ 3.7·10^4^ m.c./kg as a dose of infection with a biological agent, which caused the death of 50% of animals. According to the data obtained, the total equable *γ*-irradiation at a dose of 8.0 Gy caused the death of 50% of animals within 30 days with the development of ARD of moderate severity, which is consistent with the literature [32]. Radiation factor exposure and *P. multocida* challenge were consistent one hour apart. The experimental exposure caused the development of severe CRL with absolute lethality in animals subjected to the combined effects.

A specific feature of the course of CRBL was that it occurred earlier than the beginning of the peak period if, with isolated radiation exposure, it occurred on the 8th day. In the case of CRL, the peak occurred already on the 3d day of the postradiation period, followed by more marked clinical manifestations. A sectional study of dead animals showed that with isolated irradiation, death occurred because of pneumonia. In combined lesions, there was the development of complications—pneumonia and peritonitis—when we observed the combination with each other.

The combined effect of radiation and nonradiation factors led to an increase in the course of each of them, and the death of animals was 100%.

## 4. Conclusion

The results of the studies carried out established the minimum (half-lethal) doses of gamma rays and the causative agent of pasteurellosis, which, when combined, caused the development of combined pathology with a lethal outcome in the infected animals.

Irradiation of rabbits at a dose of 8.0 Gy infected with *Pasteurella* at a dose of *LD*_50_ (3.7·10^4^ m.c./kg) aggravated the course of the pasteurellosis process and promoted its generalization and accelerated the death of animals. The combined radiation-pasteurellosis infection ran its course rapidly, and the animals died 3–6 days after the onset of the disease. Autopsy revealed pathological signs of acute pasteurellosis: edema of the subcutaneous tissue, purulent-catarrhal bronchopneumonia, and pulmonary edema.

Bacteriological analyses of pathological material from organs and tissues from animals that died from radiation-pasteurellosis infection allowed us to isolate cultures characteristic of *P. multocida*.

## Figures and Tables

**Table 1:** Dose-dependent survival rate of rabbits infected with *Pasteurella* cultures.

Group	Dose, m.c./animal	Dead animals rate/total number of animals	Mortality rate (%)
1	1 : 10·0010^−8^	0/6	0
2	1 : 100·10^−7^	1/6	16.66
3	1 : 1000·10^−6^	2/6	33.33
4	1 : 10000·10^−5^	3/6	50
5	1 : 100000·10^−4^	5/6	83.3
6	1 : 1000000·10^−3^	6/6	100
7	1 : 10000000·10^−2^	6/6	100
8	1 : 100000000·10^−1^	6/6	100
9	1 : 1000000000·10^9^	6/6	100
10	Saline	0/6	0
11	Biological control	0/6	0

Calculation: (10 + 100)·(16.66 − 0) = 1832.6. (100 + 1000)·(33.33 − 16.66) = 18337. (1000 + 10000)·(50 − 33.33) = 183370. (10000 + 100000)·(83.3 − 50) = 3663000. (100000 + 1000000)·(100 − 83.3) = 18370000. (1000000 + 10000000)·(100 − 100) = 0. LD_50_ = 22236539.6/200 = 111182.698 m.c./animal = 111182.898/3 = 37060.89 m.c./kg (3.7·10^4^ m.c./kg).

**Table 2:** Survival rate of rabbits subjected to external *γ*-irradiation.

Group	Experience conditions	Died (day)	Average life (day)	Survival rate (%)
1	Gamma irradiation at a dose of 2.0 Gy	0	—	100
2	Gamma irradiation at a dose of 4.0 Gy	1	27	83.33
3	Gamma irradiation at a dose of 6.0 Gy	2	25	66.66
4	Gamma irradiation at a dose of 8.0 Gy	3	24	50
5	Gamma irradiation at a dose of 10.0 Gy	5	12.8	16.66
6	Gamma irradiation at a dose of 12.0 Gy	6	9.7	0
7	Biological control	0	—	100

**Table 3:** Survival rate of rabbits subjected to isolated and combined exposure to physical (gamma irradiation) and biological (infected with *P. multocida)* factors.

Group	Experience conditions	Amount of animals involved in the experiment (head)	Died (days)	Average life (days)	Survival rate, %
1	Gamma irradiation at a dose of 8.0 Gy·(*LD*_50/30_)	5	2	24	60

2	Infection with the causative agent of pasteurellosis in a half-lethal dose (3.7·10^4^ m.c./kg)	5	3	27	40

3	Combined radiation and biological action (CRBA) − gamma irradiation at a dose of 8 Gy·(*LD*_50/30_) + infection with the causative agent of pasteurellosis at a dose of 3.7·10^4^ m.c./kg·(*LD*_50/30_)	10	10	4.7	0

4	Biological control	5	0	—	100

## Data Availability

No data were used to support this study.
